# Food safety certification in urban food markets: the willingness to pay for safer meat in Peru

**DOI:** 10.1007/s12571-024-01512-6

**Published:** 2025-01-18

**Authors:** Ayako Ebata, Mauricio Espinoza, Giel Ton

**Affiliations:** 1 Institute of Development Studies, Brighton, BN1 9RE UK; 2 Grupo de Análisis del Desarrollo, Av. Almte. Miguel Grau 915, Lima, 15063 Peru; 3 Wageningen University and Research, Droevendaalsesteeg 4, 6708 PB Wageningen, the Netherlands

**Keywords:** Food safety, Wet markets, Food safety governance, Food security, LMICs

## Abstract

This paper estimates consumers' willingness to pay (WTP) for meat certified to be safe for human consumption in Peru. Citizens in low- and middle-income countries (LMICs) are increasingly concerned about the safety of food they consume. Across LMICs, urban markets remain the most important source of fresh and nutritious produce and therefore policymakers need to ensure food safety in urban markets. Much focus has been put on providing producers and supply chain actors with economic incentives to improve food safety. However, such effort has had limited effect without addressing the overall market and food safety governance. In this paper, based on an innovative policy experience from Peru, we explore if and how much consumers are willing to pay for meat sold at market stalls that are certified to provide safe meat. Peru has employed a series of economic packages to incentivise market vendors to improve their practices, and in turn increase their revenue. Our analysis based on a consumer survey across three cities in Peru reveal that consumers are willing to pay 7.1%, 5.8% and 5.3% of the average retail prices of chicken, pork and beef, respectively. This amounts to an average of about 216USD/month of extra revenue for vendors.

## Introduction

Food safety, i.e. food that does not contain harmful bacteria, viruses, parasites or chemical substances (WHO, 2024), is central to achieving food security (FAO, 2008; Zanatta et al., 2023). With rapid economic growth and subsequent changes in consumer preferences and demand for safe and nutritious food (Cicia et al., 2016; Nguyen-Viet et al., 2017), food safety has become an important issue across low- and middle-income countries (LMICs) (Grace, 2023). Regulations aiming to improve food safety – i.e. the prevalence of harmful micro-organisms or chemical elements in food – are poorly enforced across LMICs. For instance, a study on fresh fruits and vegetables in Ethiopia reports a high microbial load due to poor sanitary condition of the food stalls and hygiene practices by vendors (Kechero et al., 2019). Similarly, dairy products in China were found to be contaminated with melamine, a harmful chemical for people, leading to several death among infants (Xiu & Klein, 2010). Globally, contaminated food causes severe foodborne diseases such as diarrhoea, with major impact on cognitive and physical development and wellbeing of vulnerable adults and children (Havelaar et al., 2015a, b; WHO, 2007). While evidence is limited, food causing poisoning in people in LMICs tends to be fresh and perishable produce, such as fresh fruits and vegetables, and animal-sourced food (Grace, 2015), reflecting the challenges of supplying an increased quantity of healthy and nutrient-rich food safely.

Much focus has been put on ways to improve food safety in low-income contexts through providing producers and supply chain actors with economic incentives to improve food safety (Unnevehr, 2015). This is in a context where policymakers grapple with the need to ensure that food is safe without compromising the cost of food for vulnerable and poor people (Grace, 2015; Unnevehr, 2015). One of the tools to incentivise supply chain actors to improve food safety is creating a niche market where consumers have a willingness to pay (WTP) for food perceived and/or certified to be safe.

However, premium product prices based on higher consumer WTP have had limited effect without addressing the overall market and food safety governance (Grace, 2015). Across LMICs, the capacity of law enforcement officers is limited (Ebata et al., 2021). Without effective monitoring of food safety standards, consumers may be suspicious that food is altered along the supply chains (Soon-Sinclair et al., 2024) and lose trust in product labelling which limits their WTP (Hoffmann et al., 2019). Also, consumers may not be aware of food safety risks and therefore insufficiently informed to be willing to pay premium prices for safe products (Ortega & Tschirley, 2017). While research shows that consumers are willing to pay for presumably safe food during a food safety scandal, higher WTP for safer products is not maintained over time in a low-income setting (Hoffmann et al., 2020). This limits the economic incentives for supply chain actors to make long-term investments in improving food safety. Also, food vendors at traditional markets are often poor, lacking financial ability to make such investment (Grace, 2023).

In this paper, based on an innovative policy experience from Peru, we explore if and how much consumers are willing to pay for meat sold at market stalls that are certified to provide safe meat. We focus on wet markets, traditional markets where fresh produce are sold to the public (Petrikova et al., 2020; Zhong et al., 2020). Peru’s approach to food safety is unique because it goes beyond the dominant approach of improving stakeholders’ knowledge, attitudes and practices (KAP) (Kwoba et al., 2023), and proposes market stall-level certification, not certification at the product- or production process- (e.g. organic) levels (Unnevehr, 2022). Market stall-based certification can take advantage of trust-based relationships between consumers and vendors in judging the safety of food they purchase (Kang, 2019) where consumers commondly rely on vendors to ensure food safety at wet markets (Wertheim-Heck & Spaargaren, 2016). Therefore, in an LMIC context where wet markets remain the most critical source of food access, particularly for poor people (Liguori et al., 2022), certifying market stalls may offer a viable solution to improving food safety in LMICs. Therefore, we investigate consumer WTP for meat from certified stalls.

In Peru, the Government has implemented an incentive package for municipalities to support market vendors to fulfil food safety-related practices (MEF-Peru, 2018). The WTP estimates help to reflect on the rationale of the Peruvian policy towards certified food stalls offering better food safety conditions. Generally, improved handling practices, such as cooling and cleaning, imply higher costs for the stall owner and the WTP shows the potential price premium that they could charge to recover these costs compared to the prices of neighbouring stalls in the same market. This may also have positive spillovers in that other stalls in the same market feel obliged to improve their practices in fear of not losing clients (Dallas et al., 2019).

The rest of the article is organised as follows. In Sect. 2, we discuss food safety issues and policy effort to improve food safety in Peru before discussing our research methods in Sect. 3. In Sect. 4, we detail our statistical and econometric estimates of WTP. We finally discuss our empirical findings in the context of Peru and other LMICs and conclude in Sect. 5.

## Background: food safety in urban food markets in Peru

### Food safety evidence

At the global level, an estimated total of 600 million cases of illness were caused by foodborne diseases according to the World Health Organization’s (WHO) report in 2010 (Havelaar et al., 2015a, b). Among the reported cases, the vast majority (approximately 92%) was attributed to organisms that cause diarrhoea in people, such as *Campylobacter spp*. (ibid), commonly found in poultry species (Cardoso et al., 2021). The regional estimates by (Hoffmann et al., 2017) suggest that, in Latin America, common animal protein sources – such as beef, pork and chickens – are an important source of bacterial contamination: for instance, *Campylobacter spp.* and non-typhoidal *Salmonella* are widely found in poultry meat while Shiga-toxin producing *Escherichia coli* was detected in beef.

While Peru publishes no data that document the country’s health burden of foodborne diseases per food type (Ramirez-Hernandez et al., 2020), Ho-Palma et al. (2022) shows that *Salmonella spp.* infections from contaminated chicken and pork are a major food safety hazard in Peru. Likewise, Gonzales et al. (2023) reported the presence of Shiga toxin-producing *E. coli* 0157:H7, which can cause food- and water-borne diseases in people. Other studies document bacterial contamination in fresh fruits and vegetables. For example, fresh vegetable samples across four wet markets in Lima, the nation’s capital, demonstrated higher prevalence of *Salmonella* and *E. coli* than advised by the International Commission on Microbiological Specifications for Foods (ICMSF) (Muñoz et al., 2013). Similarly, Muñoz Ayala (2017) shows a high prevalence of *E. coli* in lettuce and spinach at markets in Lima and Pérez and Chávez (2012) attribute poor hygiene and sanitary practices by vendors in Trujillo to a high prevalence of *Listeria monocytogenes*.

### Food safety policies in Peru

Peru’s food safety policy for urban food markets is codified in Legislative Decree Nº 1062, published in 2008, which approves the “Food Safety Law”, recently reaffirmed in sanitary norm no.25 (MINSA-Peru, 2023). Multiple government entities are responsible for ensuring food safety at food markets. The authorities in charge of the control and sanitary inspection of meat in the market are the Ministry of Health through the General Directorate of Environmental Health (DIGESA), which is responsible for the safety of food intended for human consumption. The Ministry of Agriculture, through the National Agricultural Health Service (SENASA) is responsible for food safety of meat and vegetables before it enters the food markets while the food safety of the supply of fish is the responsibility of the Ministry of Production. Regional and local governments are responsible for implementing and disseminating the national food safety policy, as well as for coordinating and collaborating with the relevant authorities at the national level for the operation of the surveillance and control system. Often, municipalities have a specific department responsible for the sanitary control of food establishments, as well as for the promotion of initiatives aimed at improving food safety.

The Ministry of Finance and Planning (MEF) uses a system of result-based budget incentives to encourage local governments to implement specific policies (Ton et al., 2023). These results are known as *Metas*. Our focus is on MEF’s Metas implemented between 2018 and 2022 (MEF-Peru, 2018, 2019, 2020, 2021), which were directed to improve food safety in urban food markets (*mercados de abasto*). In 2020, the food safety aspect was complemented with social distancing requirements due to COVID-19 (Ton et al., 2023). In the pre-COVID versions of the food safety incentive programme (2017–2020), the municipalities registered all stalls and vendors in the markets, installed food safety self-monitoring committees in each market, defined sanctions and fines for markets or vendors that did not comply with basic food safety requirements, trained vendors and transporters in their municipality, and required regular inspections in order to certify the stall as a ‘healthy stall’ (*puesto saludable*).

**Fig. 1:**
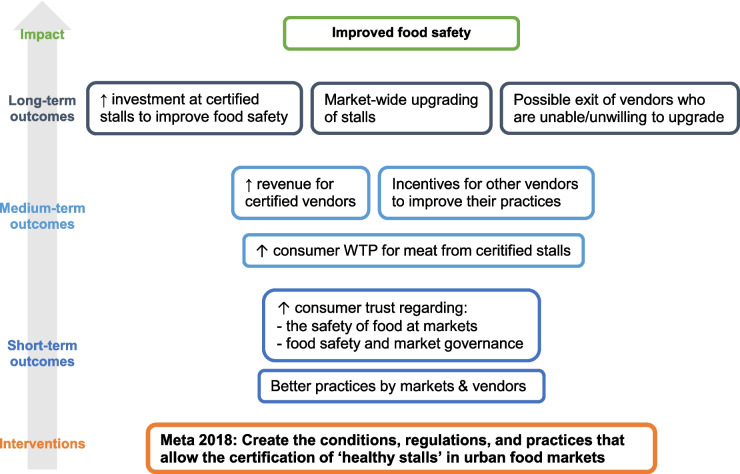
Theory of change: from certifying stalls to food safety. Source: Authors’ own

The certificate was intended as a stimulus to vendors to adopt good practices and in recognition of the additional work and investments undertaken by the stall owner. To be certified as a healthy stall, the vendors needed to comply with 75% of the required practices in two successive inspections. In Fig. 1, below, we outline the implicit theory of change toward improving food safety through certifying market vendors.

The inspection applied a list of control points across the country to evaluate the infrastructure and logistics of the markets and the individual practices of the vendors in the markets (MEF-Peru, 2018). This was followed in 2019 with a special focus on the improvement of the municipal sanitary surveillance of markets (MEF-Peru, 2019). Between 2020 and 2022, the focus on food safety was complemented with COVID-19 prevention and containment measures (MEF-Peru, 2020, 2021). Public inspections during the pandemic focused more on the mandatory social distancing measures for all stalls in the market and less on the certification of food safety conditions in individual stalls (Ton et al., 2023). In 2023, the attention returned to food safety measures (MINSA-Peru, 2023) but the earmarked funding of municipal activities under this result-based budget was discontinued. The policy to improve food safety in markets is still in place, but the interventions by the local governments to implement the policies have been reduced. Therefore, the number of certified stalls in Peru is still low.

### Are consumers willing to pay for meat from certified stalls?

Our research addresses whether consumers are willing to pay for meat from stalls certified to provide safe meat. This is distinct from previous studies, estimating WTP for *products* certified to be safe: ensuring food safety at the product-level is proven challenging particularly in low-income settings as governments lack capacity to inspect and monitor, and consumers are not always willing (or able) to pay a premium price for products certified to be safe (Hoffmann et al., 2019). Therefore, our study evaluates WTP for vendor- (i.e. market stall) level certification based on improved vendor practices. This approach may offer a viable solution to improve food safety while ensuring affordability and accessibility of nutritious food for marginalised people as wet markets in LMICs remain – and will likely remain – an important source of fresh and nutritious food for low-income people (Naguib et al., 2021; Yuan et al., 2021). While modern retail markets (i.e., supermarkets) demonstrate better hygiene (Wertheim-Heck & Raneri, 2019) and are believed to provide safer food (Rabby et al., 2021), wet markets may demonstrate comparable or better food safety performance than supermarkets (Hu et al., 2019; Ngo et al., 2021; Regalado-Pineda et al., 2020), indicating that wet markets can improve food safety with appropriate incentives and supporting mechanisms. Therefore, our study contributes to identifying ways to incentivise vendors in wet markets to adapt practices that improve food safety.

In analysing consumer WTP for healthy stalls, we employ variables that capture wider contextual factors that influence consumers’ trust in the infrastructure and management of the markets (including the certification scheme itself), local governments’ capacity to enforce food safety, and consumers’ understandings and perceptions of food safety. This makes our study novel, as most WTP studies fail to explore how these aspects of the food chain influence consumer WTP. Specifically, most studies rely on socio-economic characteristics of households – e.g. income, education, information access, number of household members, age, etc. – to explain the difference in individual WTP by consumers and not market-specific characteristics. For instance, a study in Thailand shows that consumers’ WTP for organic rice, kale and pork depends on whether a household has small children or not, lives in urban areas, and is generally healthy (Sriwaranun et al., 2015). In India, Ali and Ali (2020) show that better-educated and richer consumers indicated higher WTP for health and wellness products than their counterparts. Similarly, Chege et al. (2019) show that across East African countries, higher income, better information access and education, and having children under five years old is associated with higher WTP for improved porridge products.

We acknowledge that WTP estimates may not reflect real purchasing behaviours by consumers. In fact, a review by Hoffmann et al. (2019) shows that WTP estimates for a hypothetical product ranged between 39 and 200% of the default market price while WTP estimated based on actual purchasing behaviours were between 9 and 39%. However, an empirical evaluation of consumer purchasing behaviours is not possible within the scope of this study. To account for a possibility of overreporting WTP, we employ a double-bounded dichotomous choice (DBDC) model, which is suggested to be less biased (Britwum & Yiannaka, 2019). We detail the DBDC approach below (Sect. 3).

## Materials and methods

### Contingent valuation design

In this study, we employ a contingent valuation (CV) design to elicit consumers’ WTP for meat sold at certified stalls. CV is a standard method for assessing the monetary value of non-market goods and services, grounded in the theory of random utility maximisation (Manski, 1977). In this approach, respondents are asked the amount they are willing to pay for a product hypothetically available to them (such as in our case). The question can be asked in an open or close-ended manner, with the latter implemented through a single dichotomous question (single-bounded model) or a dichotomous question with follow-up (double-bounded model). A close-ended question is arguably preferred as it resembles how consumers make purchasing decisions in real life, thereby likely to generate a better estimate of “true” WTP than an open-ended question (Nayga et al., 2006).

We used a dichotomous question with follow-up design, or DBDC, as elicitation method (Hanemann et al., 1991). Under this framework, predetermined bids are randomly assigned to respondents, who are asked to state whether the proposed bid would be accepted (see others who used a similar method: Britwum and Yiannaka (2019); Ting et al. (2021); Wongprawmas and Canavari (2017)). If the respondent accepts the first bid, the second bid is higher than the first, but if the first bid is rejected, the second bid value is smaller than the first bid. It has been demonstrated that this approach is statistically more efficient and produces more accurate estimates compared to the conventional “single-bounded” approach (Hanemann et al., 1991). Moreover, the single-bounded approach would require larger samples to obtain accurate WTP estimates.

In this study, we asked each respondent whether they would be willing to pay a specific amount of additional Peruvian Soles (PEN)^1^ to buy 1 kg of meat from a certified market stall that ensures its meat is safe for human consumption.^2^ The initial amount of PEN proposed as an option was randomly elicited from four options: 1, 0.8, 0.5 and 0.2 PEN. These values were tested at a pilot survey to ensure that they are realistic and generate varied responses by consumers. If a respondent replied that they would be willing to pay the said amount, we asked if they would be willing to pay an even higher price for the meat. Equally, if the respondent replied that they would not be willing, we asked if they would be willing to pay a slightly lower price for the certified meat (Table 8 in the appendix depicts the amount of the initial and follow-up bids along with the sample distribution).

### Econometric model

Consumer’s WTP for certified meat was estimated using the parametric double-bounded dichotomous choice (DBDC) model. The DBDC has four possible response outcomes: (i) both answers are “yes”, (ii) a “yes” followed by a “no”, (iii) a “no” followed by a “yes”, (iv) both answers are “no”. Let us denote the likelihoods of these outcomes as ${p}^{yy}$, ${p}^{yn}$, ${p}^{ny}$, and ${p}^{nn}$. We can express these probabilities as:

1 $${p}^{yy}\left({b}_{i},{b}_{i}^{h}\right)=Prob\left({b}_{i}^{h}<{W}_{i}\right)=1-F({b}_{i}^{h};\theta)$$

2 $${p}^{yn}\left({b}_{i},{b}_{i}^{h}\right)=Prob\left({b}_{i}<{W}_{i}<{b}_{i}^{h}\right)=F\left({b}_{i}^{h};\theta \right)-F({b}_{i};\theta)$$

3 $${p}^{ny}\left({b}_{i},{b}_{i}^{l}\right)=Prob\left({b}_{i}^{l}<{W}_{i}<{b}_{i}\right)=F\left({b}_{i};\theta \right)-F({b}_{i}^{l};\theta)$$

4 $${p}^{nn}\left({b}_{i},{b}_{i}^{l}\right)=Prob\left({W}_{i}<{b}_{i}^{l}\right)=F({b}_{i}^{l};\theta)$$

where ${W}_{i}$ as the maximum willingness to pay of respondent ${^\prime}{^\prime}{i}{^\prime}{^\prime}$ for certified meat, ${b}_{i}$ the amount of the first bid, ${b}^{h}$ the amount of the second bid when the respondent answered “yes” to the first bid, and ${b}^{l}$ the amount of the second bid when the respondent answered “no” to the first bid, with ${b}_{i}^{l}<{b}_{i}<{b}_{i}^{h}$. Meanwhile, $\theta$ is a vector of parameters and $F(b;\theta$*), *$F\left({b}_{i}^{h};\theta \right)$*,* and $F\left({b}_{i}^{l};\theta \right)$ are cumulative distribution functions for the different bids.

In $(2)$ and $(3)$, the second bid allows the researcher to place both upper and lower bound on the respondent’s unobserved true WTP, while in $(1)$ and $(4)$ the second bid reflects the single (upper or lower) respondent bound (Hanemann et al., 1991). For $N$ number of respondents, the log-likelihood function for their responses can be expressed as:

5 $$Ln\left(\theta\right)=\sum\nolimits_{i=1}^N\left\{d_i^{yy}\text{ln}p^{yy}\left(b_i,b_i^h\right)+d_i^{yn}\text{ln}\;p^{yn}\left(b_i,b_i^h\right)+d_i^{ny}\text{ln}\;p^{ny}\left(b_i,b_i^l\right)+d_i^{nn}\text{ln}\;b^{nn}\left(b_i,b_i^l\right)\right\}$$

where ${d}_{i}^{yn}$, ${d}_{i}^{yn}$, ${d}_{i}^{yn}$, and ${d}_{i}^{yn}$ are binary indicator variables that equal 1 if the corresponding response outcome is observed, and 0 otherwise.

A probability distribution for $F\left({b}_{i};\theta \right)$ can be assumed to calculate the functional form of the log-likelihood equation. Then, the parameters of the model can be estimated by maximum likelihood estimation, and the expected WTP value can be derived using the delta method. The model can include covariates as explanatory variables of the WTP.

We used the build-in *doubleb* Stata-command developed by López-Feldman (2012) to estimate the WTP for certified meat. This approach assumes that ${W}_{i}$ can be modelled as a linear function, with ${u}_{i}\sim N(0,{\sigma }^{2})$. The unknown parameters $\beta$ and $\sigma$ are estimated by maximum likelihood estimation, and WTP is calculated by $E\left({W}_{i}|\widetilde{z}, \beta \right)={\widetilde{z}}{^\prime}\left[-\widehat{\alpha }/\widehat{\delta }\right]$, where $\widehat{\beta }=-\widehat{\alpha }/\widehat{\delta }$, $\widehat{\alpha }=\widehat{\beta }/\widehat{\sigma }$ (the vector of coefficients associated each one of the explanatory variables), $\widehat{\delta }=-1/\widehat{\sigma }$ (the coefficient for the variable capturing the amounts of the bid), and ${\widetilde{z}}^{\prime}$ is a vector with the values of interest for the explanatory variables (i.e., the average value, the value for a certain group).

### Study sites

We selected six urban food markets in the cities of Huaral (about 75 km from Lima), Huancayo (300 km from Lima, in the central highlands) and Tumbes (about 1,000 km from Lima near the Ecuadorian border, located on the coast). The research team purposively selected these cities as they provide diversity in key aspects of the supply chain of meat to urban fresh markets, the climate in which meat is expended, and the institutional organisation of the internal and external governance of these fresh markets. Huaral is an agricultural centre located in the coastal area and near Lima. It is an important meat production area. Huancayo is the largest urban centre in the central highlands (*Sierra*) and presents a significantly cooler climate. Tumbes is located near the Ecuadorian border on the coast and has two urban fresh markets with premises that are relatively in decline compared to the ones in Huaral and Huancayo.

As illustrated in Table 1, our sample of markets varies concerning the number of stalls, ranging from 60 to 1,000 stalls, with approximately one-fifth specialising in meat products. Table 2 shows that urban food markets (*mercados de abasto*) are more important in supplying fresh meat products to Peruvian people than stores (*bodegas*) and supermarkets. In Huaral and Huancayo, markets are the prime distribution channels for meat. In Tumbes, chicken meat is distributed primarily through stores, and pork is directly procured from farms (i.e. “Other” channel). The market share of supermarkets remains insignificant across all cities. Across the three cities, more than 60% of the fresh meat expenditure is in urban food markets and more than 90% of the households buy in these markets (Table 2).

**Table 1:** Characteristics of the markets

Market	Region	Number of stalls			Governance model	Year of creation
		Total	Operational	Sell meat		
A	Huancayo	900	600	85	Association	1988
B		1,000	1,000	114	Association	1971
C	Huaral	60	60	25	Municipal	1910
D		1,614	968	93	Association	2004
E	Tumbes	128	120	18	Municipal	2005
F		530	530	140	Association	1963

INEI-Peru (2017)

**Table 2:** Fresh meat expenditure share by retail outlets and cities

City/Meat	Market	Store	Supermarket	Street Vendor	Other	Total
*Huancayo*						
Chicken	55%	45%	0%	0%	0%	100%
Beef	85%	14%	1%	0%	0%	100%
Pork	93%	3%	1%	0%	3%	100%
*Huaral*						
Chicken	82%	18%	0%	0%	0%	100%
Beef	96%	1%	3%	0%	0%	100%
Pork	100%	0%	0%	0%	0%	100%
*Tumbes*						
Chicken	37%	60%	0%	0%	3%	100%
Beef	63%	24%	0%	0%	13%	100%
Pork	34%	7%	0%	1%	58%	100%

INEI-Peru (2022)

### Consumer survey and WTP determinants

We conducted a consumer survey between April and May 2022 to estimate WTP for meat, namely chicken, pork and beef. We randomly approached consumers in the selected markets and asked about the type of meat they had acquired within 15 days before the interview or were planning to purchase that day. Often, respondents reported purchasing multiple types of meat. As we aim to explore how the WTP differs across three meat types, we randomly assigned each consumer to one of the mentioned types of meat while ensuring that we have three similar subsample sizes per meat type. Across the six markets, this yielded 348 pork, 349 chicken and 346 beef observations. To minimise selection bias, we visited the markets in the morning and afternoon and on different days of the week.

Our questionnaire consists of a total of eight modules: 1) meat purchase and consumption habits; 2) perceptions about the meat they purchase; 3) willingness to pay for meat from certified stalls; 4) food handling practices; 5) food and nutrition security; 6) perceptions on stall infrastructure and vendor practices; 7) socio-economic characteristics; and 8) diarrhoea incidents in the previous month. Modules 1, 4, 5 and 7 address consumer characteristics and habits, while modules 2 and 6 address perceptions and attitudes about food safety.

The literature suggests that institutional factors that influence market and vendors’ food safety performance, as well as individual characteristics and risk perceptions, influence consumer WTP. We define four sets of factors (Table 3): A) individual consumer characteristics; B) perceptions and attitudes toward health, nutrition and food safety; C) the market’s infrastructural conditions; and D) contextual factors. In addition to these variables, we used dummy variables representing chicken (omitted), pork and beef to capture potential differences in perceived food safety risks, reflecting different food preparation methods and culinary purposes, prices, and frequency of consumption.

**Table 3:** Factors influencing WTP and corresponding regression variables

Factor categories	Details	Variables used
A. Observable individual characteristics of consumers	-Age, gender, education	Age; gender; education level
	-Household size and composition	# HH members; person who cooks
	-Income status and cooking conditions	Refrigerator; frequency of meat purchase; food security status
B. Individual perceptions and attitudes toward health, nutrition, and food safety	-Level of concern for food safety	Perceptions about vendor practices; risky behaviours
	-Concern for food safety conditions in the market	Concern regarding market’s conditions; distrust in meat safety at the market; rejecting meat in bad condition
	-Concern for food safety conditions in the city/area	City dummy
C. Market’s conditions	-Infrastructure	Market dummy
	-Cleanliness	
	-Organisation and governance	
D. City context and local policies	-Weather, culture and traditions	City dummy
	-Government inspections and control	
	-Transport, slaughterhouses	
	-Food safety policy (including the certification scheme)	

(Source: authors’ own)

For our econometric model, we considered as explanatory variables the factors indicated in Table 3. To select the most relevant variables within each category, we evaluated correlation among possible variables to minimise multicollinearity in conducting regression analyses. The correlation matrix can be found in the Appendix Tables 10 and 11).

## Results

### Descriptive statistics

We briefly discuss consumers’ socio-economic characteristics, meat-related practices, and their food security (for details, see Table 7 in the Appendix). Respondents reported purchasing the largest quantity of chicken (3.20 kg per month on average). For other kinds of animal protein, those in Tumbes, a coastal city, purchase more fish (2.09 kg) than other kinds of meat (i.e. pork and beef) while people in Huaral purchase the largest amount of meat and smallest amount of fish, reflecting the local culinary preferences and food availability. Most (83%) respondents have a preferred vendor in a given market. Across the three cities, food insecurity is most serious in Tumbes: 82% of respondents in Tumbes reported being concerned about lacking food because of lack of resources – such as money – while only 15% in Huaral and 57% in Huancayo reported so.

Table 4 summarises the perceived food safety, consumers’ food safety concerns and food safety-related behaviours. Respondents had a higher level of trust in the safety of chicken than beef and pork: only 7% on average responded having distrust in the safety of chicken meat compared to 16% for pork and 13% for beef. Regarding consumers’ perceptions on the importance of vendor practices, almost all (99%) in Tumbes reported that vendor practices are important to meat safety while only slightly half (55%) in Huaral and 67% in Huancayo reported so. More respondents in Tumbes (77%) are concerned about the market conditions than other two cities.

**Table 4:** Perceived food safety, food safety concerns, and consumer behaviour and attitudes (source: authors’ own)

	Total	Huancayo	Huaral	Tumbes
Perceived food safety of meat sold in the market				
Highly distrust in food safety of Chicken sold in the market (dummy)	7%	10%	5%	4%
Highly distrust in food safety of Pork sold in the market (dummy)	16%	13%	24%	9%
Highly distrust in food safety of Beef sold in the market (dummy)	13%	13%	19%	5%
Perceived food safety importance of vendors’ practices				
% of practices that considers highly important	74%	67%	55%	99%
Food safety concerns about market conditions				
Highly concerned that market’s conditions could harm food safety (dummy)	48%	48%	18%	77%
Coping strategies in response to food safety concerns				
Rejected meat that looked in bad condition (dummy)	19%	19%	22%	16%
Risk in consumer behaviour				
If sometimes or frequently eat rare fried chicken (dummy)	18%	21%	22%	11%
If sometimes or frequently eat rare hamburgers (dummy)	19%	17%	31%	8%
If sometimes or frequently eat meat in street vendors (dummy)	54%	68%	71%	21%
If sometimes or frequently eat rare beef in the house (dummy)	51%	33%	65%	56%
If sometimes or frequently use chopping table for meat and vegetables (dummy)	48%	53%	59%	31%
If does not refrigerate the meat (dummy)	20%	32%	5%	22%
% of four risk behaviours done sometimes or frequently	35%	40%	46%	18%
Preferences and attitudes concerning food safety				
Prefer clean and expensive stall than cheap and dirty stall (dummy)	85%	74%	82%	99%
Complains to vendor when the meat sold looks in bad condition (dummy)	92%	94%	84%	98%

Likewise, more respondents (99%) in Tumbes than Huancayo (74%) and Huaral (82%) reported preferring clean stalls that offer more expensive meat than dirty stalls that offer cheaper meat. Vendor practices linked to clean stalls are presented in Table 5. Most (92% on average) respondents would complain about meat in bad conditions while only 19% on average had actual experience doing so.

**Table 5:** Perceived importance of vendor practices by consumers (source: authors’ own)

Considers as highly important that vendors:	Total	Huancayo	Huaral	Tumbes
Have cleaning towels in good and clean condition (dummy)	74%	73%	50%	99%
Don’t mix the meats (dummy)	76%	62%	67%	99%
Do not place meats on a platform (dummy)	70%	60%	52%	98%
Have a stand without flies on the meats (dummy)	84%	77%	74%	100%
Have running water (dummy)	73%	71%	48%	99%
Display the meat on counters with a cold chain (dummy)	70%	59%	54%	99%
Have refrigeration chambers (dummy)	72%	58%	60%	99%
Use clean hooks to hang display meats (dummy)	78%	74%	62%	99%
Have equipment/utensils made of stainless material (dummy)	78%	70%	63%	100%
Have solid waste containers with lids (dummy)	75%	77%	49%	99%
Use proper chopping board (not trunk) (dummy)	63%	61%	28%	99%

Regarding risky practices, roughly 20% of respondents in Huancayo and Huaral reported sometimes eating fried chicken that is not well-cooked through, while only 11% in Tumbes reported doing so. More respondents in Huaral reported sometimes eating rare hamburgers and beef (31% and 65%, respectively) than those in Huancayo (17% and 33%, respectively) and Tumbes (8% and 56%). More people eat meat at street vendors in Huancayo and Huaral than Tumbes, although we do not have data to conclude that food by street vendors is more likely to be contaminated than other food outlets such as restaurants. 53% and 59% of respondents in Huancayo and Huaral, respectively, use the same chopping board for meat and vegetables while only 31% reported doing so in Tumbes. Most people refrigerate their meat upon purchase in Huaral (95%) but 32% in Huancayo and 22% in Tumbes reported not doing so. In Tumbes, this is likely due to the high poverty level while in Huancayo, the climate tends to be cool and therefore people may not perceive the need to own a refrigerator.

### WTP estimates

**Fig. 2:**
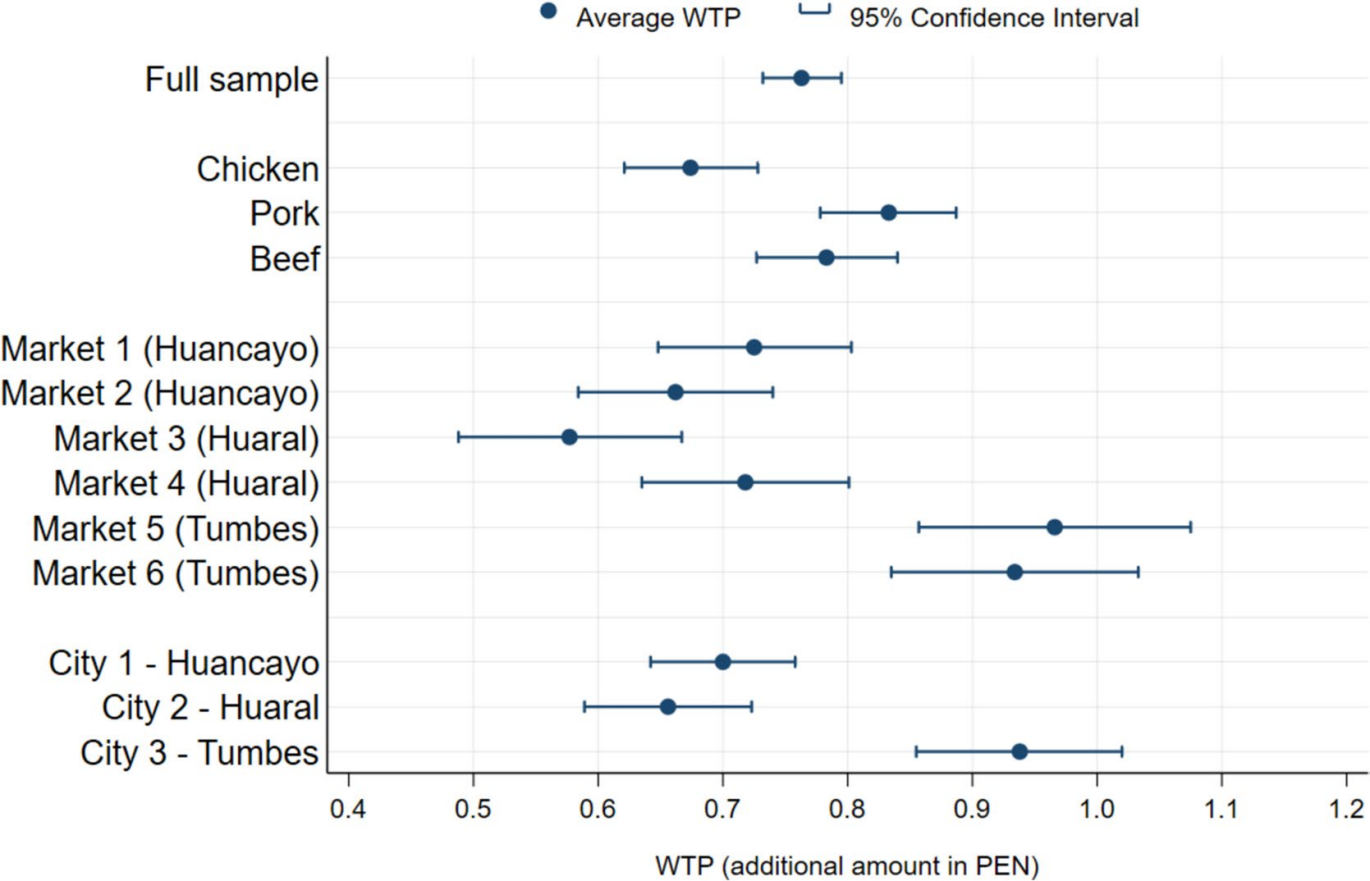
WTP for meat from a food safety certified stall, in Peruvian Soles (additional PEN per kilo) (source: authors’ own)

As discussed in Sect. 3.1, we employ the double-bounded model to calculate the WTP for consumers across meat types, cities, and markets where the data was collected (Fig. 2). These figures capture the additional amount of PEN that consumers are willing to pay for one kilogram of meat sold at certified stalls. In other words, we assume that consumers are willing to pay the current market price for meat from stalls that are not certified. The average WTP for chicken is the lowest at 0.68 PEN (more than the current market rate) and that for pork is the highest at 0.83 PEN. Consumers in Tumbes reported the highest average WTP (0.94 PEN), with Huaral reporting the lowest (0.66 PEN). Despite certain contrasts, the average WTP remains similar across markets within the same city, underscoring the influence of environmental and local policy dimensions in explaining WTP.

### WTP determinants

In this section, we examine a wider set of factors contributing to individual WTP differences among consumers (Table 6). All estimated models incorporate consumer and household characteristics, alongside fixed effects for meat-type and city.^3^ The models differ concerning the inclusion of five food safety dimensions: (i) perceived importance of vendor practices linked to food safety, (ii) food safety concerns about market conditions, (iii) consumer behaviour risk, (iv) perceived food safety of meat sold in the market, and (v) attitude concerning food safety. Models (1) to (5) include each of these dimensions separately, while model (6) includes the five dimensions altogether. For ease of interpretation, all variables were standardised to have a mean of zero and a standard deviation of one, enabling us to identify and compare the most important factors affecting WTP among consumers.

**Table 6:** Double-bounded model estimation of WTP for meat from a certified stall (additional PEN per kilo)

VARIABLES	Model (1)	Model (2)	Model (3)	Model (4)	Model (5)	Model (6)
Number of vendor’s FS practices considered as important (1–11)	0.162***					0.135***
	(0.021)					(0.022)
Highly concerned that market’s conditions could harm FS (dummy)		0.110***				0.066***
		(0.019)				(0.019)
Number of risk behaviours practiced sometimes or frequently (1–4)			−0.097***			−0.085***
			(0.018)			(0.018)
Distrust in the safety of meat sell in the market (dummy)				−0.051***		−0.002
				(0.018)		(0.018)
Rejected meat that looked in bad condition (dummy)					−0.010	0.006
					(0.017)	(0.016)
Age	0.019	0.020	0.007	0.017	0.021	0.008
	(0.017)	(0.018)	(0.018)	(0.018)	(0.018)	(0.017)
Female (dummy)	0.030	0.031	0.032	0.038*	0.037*	0.024
	(0.021)	(0.021)	(0.021)	(0.021)	(0.021)	(0.020)
Level of education (1 = None, 8 = Postgraduate)	0.045**	0.052***	0.053***	0.062***	0.060***	0.039**
	(0.019)	(0.020)	(0.020)	(0.020)	(0.020)	(0.019)
Refrigerator (dummy)	0.026	0.039**	0.043**	0.045**	0.049***	0.017
	(0.018)	(0.018)	(0.017)	(0.018)	(0.018)	(0.017)
Household size	0.021	0.022	0.027	0.023	0.029	0.017
	(0.018)	(0.018)	(0.018)	(0.019)	(0.018)	(0.018)
Person who cooks (dummy)	0.032	0.028	0.028	0.028	0.030	0.029
	(0.021)	(0.021)	(0.021)	(0.022)	(0.022)	(0.020)
Frequency of meat purchase at the market (times per month)	−0.029	−0.023	−0.021	−0.024	−0.024	−0.025
	(0.020)	(0.020)	(0.020)	(0.021)	(0.021)	(0.020)
Food insecurity concerning meat access (dummy)	−0.054***	−0.063***	−0.063***	−0.062***	−0.063***	−0.055***
	(0.019)	(0.020)	(0.020)	(0.020)	(0.020)	(0.019)
Constant	0.764***	0.767***	0.766***	0.767***	0.767***	0.763***
	(0.016)	(0.017)	(0.016)	(0.017)	(0.017)	(0.016)
Sigma	0.456***	0.466***	0.462***	0.473***	0.474***	0.445***
	(0.015)	(0.015)	(0.015)	(0.016)	(0.016)	(0.015)
Observations	1,042	1,042	1,042	1,039	1,042	1,039
Market fixed effects	Yes	Yes	Yes	Yes	Yes	Yes
Meat type fixed effects	Yes	Yes	Yes	Yes	Yes	Yes
Wald chi2	298	265	266	239	234	338
Log-Likelihood	−1,330.0	−1,344.3	−1,347.2	−1,354.4	−1,361.0	−1,308.7

Standard errors in parentheses. *** *p*< 0.01, ** *p*< 0.05, * *p*< 0.1

The results, detailed in Table 6, indicate that the most important factor is the perceived importance of vendors’ practices linked to food safety. Consumers who express greater concern exhibit a higher WTP. A one standard deviation increase in the number of vendors’ practices influencing food safety considered important by consumers results in a WTP increase of over 0.13 PEN (18% of the average WTP). This finding underscores the critical role consumers ascribe to these actors in ensuring meat safety. Consumers also attach importance to the conditions and infrastructure of the markets. A one-standard-deviation increase in the percentage of consumers highly concerned that market conditions could jeopardise the food safety of meat leads to a WTP increase of around 0.07 PEN (9%). Furthermore, consumers with risky behaviours concerning food safety are willing to pay less for food from certified stalls. This finding indicates that those less concerned with food safety are unlikely to be willing to pay a high premium price for certified food, which is intuitive. This finding aligns with the observed negative association between consumers’ distrust in the safety of meat sold in the market and their WTP (Model 4).

Consumers’ perceptions regarding vendor practices and market conditions are more influential to their WTP than their individual attributes and economic status. Among these factors, only the level of education, ownership of a refrigerator, and food insecurity status are significant predictors of consumers’ WTP. Higher WTP is associated with consumers with higher education levels, ownership of assets (i.e., refrigerator), and better food security.

## Discussion and conclusions

Our findings and wider literature (FAO, 2023; OECD, 2021) suggest that markets will remain the main source of fresh and nutritious food for people in Peru and other LMICs. Food safety control in markets is key to preventing gastrointestinal infection, which can cause severe physical and cognitive issues (Grace et al., 2018). Food safety norms are difficult to enforce, and, as the COVID-19 pandemic showed (Ton et al., 2023), markets cannot simply be closed down because of food safety concerns. Peru’s effort to improve food safety in markets through result-based budget allocations to local governments (*Metas*) is a unique tool that aimed to create a group of certified market vendors that have improved practices linked to food safety and could attract consumers willing to pay more for the meat.

We find that, on average, consumers are willing to pay an additional 0.76 PEN for meat (i.e. chicken, pork and beef) from certified stalls (Fig. 2), and the WTP estimates range between 0.5 to 0.9 PEN per kg, depending on the type of meat, city or market considered. This is not trivial: it corresponds to 7.1%, 5.8% and 5.3% of the average retail prices of chicken, pork and beef, respectively (see Appendix Table 9). A survey of meat vendors across the six markets in 2021 (see Appendix Table 11) indicates that vendors’ daily sales of fresh meat range between 30 kg (beef) and 60 kg (chicken). Consequently, if certified, vendors could earn an average of approximately 800 PEN (approximately 216 USD) monthly.^4^ This estimate does not account for a possibility that increased price would decrease consumption. However, Peru’s per capita meat production – particularly beef and pork – is significantly lower than other countries with industrial production systems (FAO, 2024).^5^ Therefore, we expect that the production and consumption of animal protein will further increase, and market vendors will continue to play a major role in providing Peruvian people with meat. This substantial additional income for market vendors could enable vendors to make and maintain significant investments in enhancing food safety in their stalls. Thus, the policy’s rationale to advance towards the certification of stalls in the market (Fig. 1) seems sound and plausible.

Our analysis also indicates that the WTP differs across cities and markets. While these differences could be explained by a wide variety of factors specific to the cities and markets, our findings suggest that market infrastructure and market governance – both within the market space and by the municipal and food safety agencies – critically influence the WTP. For instance, markets in Tumbes had the poorest conditions among the 6 markets we worked in. This likely led to consumers in Tumbes most concerned about, and therefore most willing to pay for, food safety. This is despite that people in Tumbes were the poorest compared to Huaral and Huancayo. These institutional and infrastructural factors are out of the sphere of direct influence of individual vendors, market owners, and market associations, and therefore local governments need a strategy to ensure investment in these areas of collective interest. The Peruvian policy of earmarked funding is an innovative way of supporting this in a context specific manner as each authority is able to target key areas of governance and infrastructure development.

In conclusion, improving food safety in LMICs will require a combination of approaches that improve the individual vendor, market governance, and institutional supervision capacities and willingness to invest in food safety environments like urban food markets (OECD, 2021; WHO, 2022). Our study shows that certification of individual stalls in urban markets can be a vital instrument to do so, as it can potentially increase revenue by market vendors that invest in practices and technologies linked to food safety. Such an approach may be more effective than the “command and control” approach (OECD, 2021) in LMICs where the informal sector dominates (Ton et al., 2023), and the authorities lack capacity to regulate (Ebata et al., 2021). Certification of market vendors, therefore, takes account of the technical, social and economic aspects of food safety improvement (Grace et al., 2018) in resource-poor and informal settings.

## Data Availability

The data used for this publication can be shared upon request.

## Appendix

see Tables 7, 8, 9, 10, 11 and 12

**Table 7:** Consumer socio-economic characteristics and meat purchasing practices

	Total	Huancayo	Huaral	Tumbes
Individual and household characteristics				
Informant’s female (dummy)	83%	75%	85%	89%
Informant’s age	42.7	42.7	41.7	43.7
Informant attained at least seondary school (dummy)	81%	95%	78%	71%
Informant attained undergraduate studies (dummy)	37%	55%	32%	23%
Family size	4.2	3.7	4.2	4.8
Person who cooks in the house (dummy)	79%	67%	78%	92%
Meat purchasing practices				
Times per month that buy meat at the market	10.7	9.0	7.9	15.2
Times per month that eat meat at the household	23.1	19.1	23.8	26.4
Purchased chicken at the market in the past 15 days	90%	73%	99%	100%
Purchased pork at the market in the past 15 days	63%	54%	62%	72%
Purchased beef at the market in the past 15 days	79%	62%	82%	92%
Purchased fish at the market in the past 15 days	65%	43%	59%	95%
Purchased other meat at the market in the past 15 days	27%	40%	26%	14%
Monthly per capita Kg. of chicken purchased at the market	3.20	2.25	4.11	2.99
Monthly per capita Kg. of pork purchased at the market	1.34	1.48	1.59	1.02
Monthly per capita Kg. of beef purchased at the market	1.27	1.20	1.68	0.94
Monthly per capita Kg. of fish purchased at the market	1.56	0.81	1.26	2.09
Monthly per capita Kg. of other meat purchased at the market	1.00	0.91	1.14	1.03
Has a regular vendor (*casero*)(dummy)	83%	75%	90%	85%
Food insecurity				
Highly concerned on food security due to lack of resources	51%	57%	15%	82%
Did not eat at least one basic meal due to lack of resources	21%	3%	7%	54%
Did not purchased meat due to lack of resources	31%	26%	9%	60%

**Table 8:** Bid schemes and distribution of response outcomes

Bids	Initial Bid (PEN)	Decreased follow-up bid (PEN)	Increased follow-up bid (PEN)	Response outcomes for first and second bid				Obs
				YY	YN	NY	NN	
1	1.00	0.80	1.50	24%	33%	9%	33%	269
2	0.80	0.50	1.00	23%	22%	27%	29%	258
3	0.50	0.20	0.80	29%	38%	23%	10%	257
4	0.20	0.10	0.50	46%	29%	15%	10%	259

YY stands for % of respondents who answer “yes” in both bids, YN for % of respondents who answer “yes” in the first bid and “no” in the second bid, NY for % of respondents who answer “no” in the first bid and “yes” in the second bid, and NN for % of respondents who answer “no” in both bids

**Table 9:** Reported retail prices of different meat products and elicited WTP

	National household survey 2021					
Carne	Mean	Median	Obs	WTP	% mean	% median
Whole chicken	9.7	9.5	8,921	0.67	7.1%	7.2%
Pork meat	14.5	14.0	3,262	0.83	5.8%	6.0%
Beef meat	14.5	14.0	2,788	0.78	5.3%	5.5%

INEI-Peru (2021) and authors’ own

**Table 10:** Correlation matrix across selected WTP drivers

	(1)	(2)	(3)	(4)	(5)
(1) % of practices that are considered as highly important	1				
(2) % of four risk behaviours done sometimes or frequently	-0.31	1			
(3) Highly concerned that market conditions could harm food safety	0.51	-0.26	1		
(4) High distrust in the safety of meat sold in the market	-0.31	0.19	-0.30	1	
(5) Rejected meat that looked in bad condition	-0.19	-0.03	0.00	0.12	1

**Table 11:** Market’s vendors reported daily sales of meat (kg of meat, May 2021), N = 213

City	Beef	Pork	Chicken
Huancayo	24.8	40.7	65.9
Huaral	36.8	48.2	74.7
Tumbes	13.0	7.4	17.5
Total	26.7	38.6	62.3
Obs (vendors)	99	47	77

Vendor’s survey (Unpublished, collected May 2021)

**Table 12:** Consumers’ WTP lower bound according to different thresholds

	Initial bid			
	PEN 0.20	PEN 0.50	PEN 0.80	PEN 1.00
WTP more than 0.10 PEN	90%			
WTP more than 0.20 PEN	75%	90%		
WTP more than 0.50 PEN	46%	67%	71%	
WTP more than 0.80 PEN		29%	45%	67%
WTP more than 1.00 PEN			23%	58%
WTP more than 1.50 PEN				24%
Observations (consumers)	259	257	258	269

## Footnotes

1 1USD = approximately 3.75 Peruvian Soles as of April and May, 2022.

2 The exact phrase in Spanish is: ¿Usted estaría dispuesto a pagar X sol adicional por kilo de carne de (chancho, pollo o res) que se venda en un puesto que tiene un sello que garantiza que su consumo es seguro para la salud?

3 The results are nearly identical if we include market fixed effects instead.

4 As a reference, the monthly minimum wage in Peru was 930 PEN in 2021.

5 For instance, Peru produced 5.2 tons/capita of pork in 2021 compared to Argentina (15 tons/capita), Brazil (23 tons/capita), and USA (37 tons/capita). Likewise, Peru’s per capita beef production was at 5.6 tons in 2021 compared to 66 in Argentina, 45 in Brazil, and 38 in USA.

## References

[CR1] Ali, T., & Ali, J. (2020). Factors affecting the consumers’ willingness to pay for health and wellness food products. *Journal of Agriculture and Food Research,**2*, 100076. 10.1016/J.JAFR.2020.100076

[CR2] Britwum, K., & Yiannaka, A. (2019). Consumer willingness to pay for food safety interventions: The role of message framing and issue involvement. *Food Policy*, *86*. 10.1016/j.foodpol.2019.05.009

[CR3] Cardoso, M. J., Ferreira, V., Truninger, M., Maia, R., & Teixeira, P. (2021). Cross-contamination events of Campylobacter spp. in domestic kitchens associated with consumer handling practices of raw poultry. *International Journal of Food Microbiology*, *338*. 10.1016/J.IJFOODMICRO.2020.108984 10.1016/j.ijfoodmicro.2020.108984 33277046

[CR4] Cicia, G., Caracciolo, F., Cembalo, L., Del Giudice, T., Grunert, K. G., Krystallis, A., Lombardi, P., & Zhou, Y. (2016). Food safety concerns in urban China: Consumer preferences for pig process attributes. *Food Control*, *60*, 166–173. 10.1016/j.foodcont.2015.07.012

[CR5] Chege, C. G. K., Sibiko, K. W., Wanyama, R., Jager, M., & Birachi, E. (2019). Are consumers at the base of the pyramid willing to pay for nutritious foods? *Food Policy,**87*, 101745. 10.1016/J.FOODPOL.2019.101745

[CR6] Dallas, M. P., Ponte, S., & Sturgeon, T. J. (2019). Power in global value chains. *Review of International Political Economy,**26*(4), 666–694. 10.1080/09692290.2019.1608284

[CR7] Ebata, A., Thorpe, J., Islam, A., Sultana, S., & Mbuya, M. N. N. (2021). Understanding drivers of private-sector compliance to large-scale food fortification: A case study on edible oil value chains in Bangladesh. *Food Policy,**104*, 102127. 10.1016/j.foodpol.2021.102127 34720342 10.1016/j.foodpol.2021.102127 PMC8546401

[CR8] FAO. (2008). An Introduction to Basic Concepts of Food Security. *In Social Indicators Research*. 10.1007/s11524-010-9491-z

[CR9] FAO. (2023). *Understanding the potential for territorial markets to promote healthy diets Evidence from Lebanon*. 10.4060/cc7353en

[CR10] FAO. (2024). *FAOSTAT*. http://faostat.fao.org/beta/en/#home. Accessed 24 Nov 2024.

[CR11] Gonzales, B. L., Andrade, D. A., Valdivia, C. A., Ho-Palma, A. C., Munguia, A., Yucra, D., Escobedo, M., Crotta, M., Limon, G., Gonzalez, A., Guitian, J., & Gonzales-Gustavson, E. (2023). Detection and Isolation of Escherichia coli O157: H7 in Beef from Food Markets and Fecal Samples of Dairy Calves in the Peruvian Central Highlands. *The American Journal of Tropical Medicine and Hygiene,**109*(3), 568–570. 10.4269/AJTMH.23-0181 37487566 10.4269/ajtmh.23-0181 PMC10484278

[CR12] Grace, D. (2015). Food safety in low and middle income countries. *International Journal of Environmental Research and Public Health,**12*(9), 10490–10507. 10.3390/ijerph120910490 26343693 10.3390/ijerph120910490 PMC4586623

[CR13] Grace, D., Alonso, S., Mutua, F., Roesel, K., Lindahl, J., & Amenu, K. (2018). *Food safety investment expert advice: Burkina Faso, Ethiopia, Nigeria*.www.ilri.org. Accessed 12 Dec 2023.

[CR14] Grace, D. (2023). Burden of foodborne disease in low-income and middle-income countries and opportunities for scaling food safety interventions. In *Food Security*. Springer Science and Business Media B.V. 10.1007/s12571-023-01391-3

[CR15] Hanemann, M., Loomis, J., & Kanninen, B. (1991). Statistical efficiency of double-bounded dichotomous choice contingent valuation. *American Journal of Agricultural Economics,**73*(4), 1255–1263. 10.2307/1242453

[CR16] Havelaar, A. H., Kirk, M. D., Torgerson, P. R., Gibb, H. J., Hald, T., Lake, R. J., Praet, N., Bellinger, D. C., de Silva, N. R., Gargouri, N., Speybroeck, N., Cawthorne, A., Mathers, C., Stein, C., Angulo, F. J., Devleesschauwer, B., Adegoke, G. O., Afshari, R., Alasfoor, D., …& Zeilmaker, M. (2015a). World health organization global estimates and regional comparisons of the burden of foodborne disease in 2010. *PLoS Medicine*, *12*(12), 1–23. 10.1371/journal.pmed.1001923 10.1371/journal.pmed.1001923 PMC4668832 26633896

[CR17] Havelaar, A. H., Kirk, M. D., Torgerson, P. R., Gibb, H. J., Hald, T., Lake, R. J., Praet, N., Bellinger, D. C., de Silva, N. R., Gargouri, N., Speybroeck, N., Cawthorne, A., Mathers, C., Stein, C., Angulo, F. J., Devleesschauwer, B., Adegoke, G. O., Afshari, R., Alasfoor, D., …& Zeilmaker, M. (2015b). World health organization global estimates and regional comparisons of the burden of foodborne disease in 2010. *PLoS Medicine*, 12(12), 1–23. 10.1371/journal.pmed.1001923 10.1371/journal.pmed.1001923 PMC4668832 26633896

[CR18] Hoffmann, S., Devleesschauwer, B., Aspinall, W., Cooke, R., Corrigan, T., Havelaar, A., Angulo, F., Gibb, H., Kirk, M., Lake, R., Speybroeck, N., Torgerson, P., & Hald, T. (2017). Attribution of global foodborne disease to specific foods: Findings from a world health organization structured expert elicitation. *PLoS ONE,**12*(9), e0183641. 10.1371/JOURNAL.PONE.0183641 28910293 10.1371/journal.pone.0183641 PMC5598938

[CR19] Hoffmann, V., Moser, C., & Saak, A. (2019). Food safety in low and middle-income countries: The evidence through an economic lens. *World Development,**123*, 104611. 10.1016/j.worlddev.2019.104611

[CR20] Hoffmann, V., Moser, C. M., & Herrman, T. J. (2020). *Demand for Aflatoxin-Safe Maize in Kenya: Dynamic Response to Price and Advertising*. 10.1111/ajae.12093

[CR21] Ho-Palma, A. C., Gonzales-Gustavson, E., Quispe, E., Crotta, M., Nunney, E., Limon, G., Andrade-Mogrovejo, D., Ton, G., Espinoza, M., Fort, R., Pastor, J., Yabar, E., Solis, J., Gil, A. I., Ordoñez Ibargüen, L., Gonzalez, A., & Guitian, J. (2022). Salmonella in chicken and pork meat are likely to be a major contributor to foodborne illness in Peru. *SSRN Electronic Journal*. 10.2139/SSRN.4226470

[CR22] Hu, D. W., Liu, C. X., Zhao, H. B., Ren, D. X., Zheng, X. D., & Chen, W. (2019). Systematic study of the quality and safety of chilled pork from wet markets, supermarkets, and online markets in China. *Journal of Zhejiang University. Science. B,**20*(1), 95. 10.1631/JZUS.B1800273 30614233 10.1631/jzus.B1800273 PMC6331336

[CR23] INEI-Peru. (2017)*. Censo nacional de mercados de abasto. 2016: resultados a nivel nacional [National census of food markets 2016: national-level results]. *Instituto Nacional de Estadística e Informática.

[CR24] INEI-Peru. (2021). *Encuesta nacional de hogares 2021 [National household survey 2018]. *Instituto Nacional de Estadística e Informática.

[CR25] INEI-Peru. (2022). *Encuesta nacional de hogares 2022 [National household survey 2018]. *Instituto Nacional de Estadística e Informática.

[CR26] Kang, Y. (2019). Food safety governance in China: Change and continuity. *Food Control,**106*, 106752. 10.1016/J.FOODCONT.2019.106752

[CR27] Kechero, F. K., Baye, K., Tefera, A. T., & Tessema, T. S. (2019). Bacteriological quality of commonly consumed fruit juices and vegetable salads sold in some fruit juice houses in Addis Ababa. *Ethiopia. Journal of Food Safety,**39*(1), e12563. 10.1111/JFS.12563

[CR28] Kwoba, E., Oduori, D. O., Lambertini, E., Thomas, L. F., Grace, D., & Mutua, F. (2023). Food safety interventions in low- and middle-income countries in Asia: A systematic review. *In Zoonoses and Public Health,**70*(3), 187–200. 10.1111/zph.13028. John Wiley and Sons Inc. 10.1111/zph.13028 36718488

[CR29] Liguori, J., Trübswasser, U., Pradeilles, R., Le Port, A., Landais, E., Talsma, E. F., Lundy, M., Béné, C., Bricas, N., Laar, A., Amiot, M. J., Brouwer, I. D., & Holdsworth, M. (2022). How do food safety concerns affect consumer behaviors and diets in low- and middle-income countries? A systematic review. *Global Food Security*, *32*(July 2021). 10.1016/j.gfs.2021.100606

[CR30] López-Feldman, A. (2012). *Introduction to contingent valuation using Stata Introduction to Contingent Valuation using Stata (MPRA Paper No. 41018)*. https://mpra.ub.uni-muenchen.de/41018

[CR31] Manski, C. F. (1977). The Structure of Random Utility Models. *Theory and Decision*, *8*(3), 229. https://www.proquest.com/openview/7acf07ef00e4d7b837b4de87994aed40/1?pq-origsite=gscholar&cbl=1818302. Accessed 8 Nov 2023.

[CR32] MEF-Peru. (2018). *Meta 20: Certificacion de Puestos de Venta Saludables de Alimentos Agropecuarios Primarios y Piensos, en Mercados de Abastos.*

[CR33] MEF-Peru. (2019). *Meta 6: Mejora de la vigilancia sanitaria municipal de puestos de venta saludables de alimentos agropecuarios primarios y piensos [Target 6: Improvement of municipal health surveillance of healthy stalls that sell primary agricultural products and animal feed]. *Ministerio de Economía y Finanzas.

[CR34] MEF-Peru. (2020). *Meta 1: Regulación del funcionamiento de los mercados de abastos para la prevención y contención del COVID-19 [Target 1: Regulation on the functioning of food markets to prevent and contain COVID-19]. *Ministerio de Economía & Ministerio de Producción.

[CR35] MEF-Peru. (2021).* Meta 6: Regulación del funcionamiento de los mercados de abastos para la prevención y contención del COVID-19 [Target 6: Regulation on the functioning of food markets to prevent and contain COVID-19]. *Ministerio de Economía & Ministerio de Salud*.*

[CR36] MINSA-Peru. (2023). *Reglamento Sanitario de Funcionamiento de Mercados de Abasto. N° 282-2003-SA/DM [Sanitary food market regulation]. *El Peruano.

[CR37] Muñoz, S. J., Vilca, M. L., Ramos, D. D., & Lucas, J. L. (2013). Frecuencia de enterobacterias en verduras frescas de consumo crudoexpendidas en cuatro mercados de Lima, Perú. *Revista de Investigaciones Veterinarias Del Perú,**24*(3), 300–306. 10.15381/rivep.v24i3.2578

[CR38] Muñoz Ayala, M. D. (2017). *Escherichia coli O157:H7 en hortalizas de fundos agrícolas en la periferia de la ciudad de Lima - Perú*. Universidad Nacional Mayor de San Marcos.

[CR39] Naguib, M. M., Li, R., Ling, J., Grace, D., Nguyen-Viet, H., & Lindahl, J. F. (2021). Live and wet markets: Food access versus the risk of disease emergence. *Trends in Microbiology*, *29*(7). 10.1016/j.tim.2021.02.007 10.1016/j.tim.2021.02.007 PMC9189808 33712334

[CR40] Nayga, R. M., Woodward, R., & Aiew, W. (2006). Willingness to pay for reduced risk of foodborne illness: A nonhypothetical field experiment. *Canadian Journal of Agricultural Economics,**54*, 461–475.

[CR41] Ngo, H. H. T., Nguyen-Thanh, L., Pham-Duc, P., Dang-Xuan, S., Le-Thi, H., Denis-Robichaud, J., Nguyen-Viet, H., Le, T. T. H., Grace, D., & Unger, F. (2021). Microbial contamination and associated risk factors in retailed pork from key value chains in Northern Vietnam. *International Journal of Food Microbiology,**346*, 109163. 10.1016/J.IJFOODMICRO.2021.109163 33798966 10.1016/j.ijfoodmicro.2021.109163

[CR42] Nguyen-Viet, H., Tuyet-Hanh, T. T., Unger, F., Dang-Xuan, S., & Grace, D. (2017). Food safety in Vietnam: Where we are at and what we can learn from international experiences. *Infectious Diseases of Poverty*, *6*(1). 10.1186/s40249-017-0249-7 10.1186/s40249-017-0249-7 PMC5314466 28209208

[CR43] OECD. (2021). Food safety challenges, informal markets, and their role in the crisis. In *Improving Regulatory Delivery in Food Safety: Mitigating Old and New Risks, and Fostering Recovery*. 10.1787/d1a4faad-en

[CR44] Ortega, D. L., & Tschirley, D. L. (2017). Demand for food safety in emerging and developing countries: A research agenda for Asia and Sub-Saharan Africa. *Journal of Agribusiness in Developing and Emerging Economies,**7*(1), 21–34. 10.1108/JADEE-12-2014-0045

[CR45] Pérez, E., & Chávez, M. (2012). Frecuencia de Listeria monocytogenes en tomate, zanahoria, espinaca, lechuga y rabanito, expendidos en mercados de Trujillo, Perú. *Revista Ciencia y Tecnología*, *8*(22), 11–21. https://revistas.unitru.edu.pe/index.php/PGM/article/view/183. Accessed 15 Jan 2024.

[CR46] Petrikova, I., Cole, J., & Farlow, A. (2020). COVID-19, wet markets, and planetary health. *The Lancet Planetary Health,**4*(6), e213–e214. 10.1016/S2542-5196(20)30122-4 32559435 10.1016/S2542-5196(20)30122-4 PMC7832206

[CR47] Rabby, M. R. I., Shah, S. T., Miah, M. I., Islam, M. S., Khan, M. A. S., Rahman, M. S., & Malek, M. A. (2021). Comparative analysis of bacteriological hazards and prevalence of Salmonella in poultry-meat retailed in wet- and super-markets in Dhaka city, Bangladesh. *Journal of Agriculture and Food Research,**6*, 100224. 10.1016/J.JAFR.2021.100224

[CR48] Ramirez-Hernandez, A., Galagarza, O. A., Álvarez Rodriguez, M. V., Pachari Vera, E., del Valdez Ortiz, M. C., Deering, A. J., & Oliver, H. F. (2020). Food safety in Peru: A review of fresh produce production and challenges in the public health system. *Comprehensive Reviews in Food Science and Food Safety,**19*(6), 3323–3342. 10.1111/1541-4337.12647 33337060 10.1111/1541-4337.12647

[CR49] Regalado-Pineda, I. D., Rodarte-Medina, R., Resendiz-Nava, C. N., Saenz-Garcia, C. E., Castañeda-Serrano, P., & Nava, G. M. (2020). Three-year longitudinal study: Prevalence of Salmonella enterica in chicken meat is higher in supermarkets than wet markets from Mexico. *Foods,**9*(3), 264. 10.3390/FOODS9030264 32121659 10.3390/foods9030264 PMC7143798

[CR50] Soon-Sinclair, J. M., Ha, T. M., Vanany, I., Limon, M. R., Sirichokchatchawan, W., Abdul Wahab, I. R., Hamdan, R. H., & Jamaludin, M. H. (2024). Consumers’ perceptions of food fraud in selected Southeast Asian countries: A cross sectional study. *Food Security,**16*(1), 65–77. 10.1007/S12571-023-01406-Z/TABLES/4

[CR51] Sriwaranun, Y., Gan, C., Lee, M., & Cohen, D. A. (2015). Consumers’ willingness to pay for organic products in Thailand. *International Journal of Social Economics,**42*(5), 480–510. 10.1108/IJSE-09-2013-0204

[CR52] Ting, C. T., Huang, Y. S., Lin, C. T., & Hsieh, Y. (2021). Measuring consumer’ willingness to pay for food safety certification labels of packaged rice. *AIMS Agriculture and Food,**6*(4), 1000–1010. 10.3934/AGRFOOD.2021060

[CR53] Ton, G., Espinoza, M., & Fort, R. (2023). COVID policy and urban food markets in Peru: Governance and compliance. *The Journal of Development Studies,**59*(6), 854–872. 10.1080/00220388.2023.2178303

[CR54] Unnevehr, L. (2015). Food safety in developing countries: Moving beyond exports. *In Global Food Security,**4*, 24–29. 10.1016/j.gfs.2014.12.001. Elsevier B.V.

[CR55] Unnevehr, L. J. (2022). Addressing food safety challenges in rapidly developing food systems. *Agricultural Economics (United Kingdom),**53*(4), 529–539. 10.1111/agec.12724

[CR56] Wertheim-Heck, S. C. O., & Raneri, J. E. (2019). A cross-disciplinary mixed-method approach to understand how food retail environment transformations influence food choice and intake among the urban poor: Experiences from Vietnam. *Appetite,**142*(April), 104370. 10.1016/j.appet.2019.104370 31310835 10.1016/j.appet.2019.104370 PMC6739597

[CR57] Wertheim-Heck, S. C. O., & Spaargaren, G. (2016). Shifting configurations of shopping practices and food safety dynamics in Hanoi, Vietnam: A historical analysis. *Agriculture and Human Values,**33*(3), 655–671. 10.1007/s10460-015-9645-4

[CR58] WHO. (2007). *ESTIMATES OF THE GLOBAL BURDEN OF FOODBORNE DISEASES*. www.who.int. Accessed 20 Mar 2024.

[CR59] WHO. (2022). WHO global strategy for food safety: reducing public health risks associated with the sale of live wild animals of mammalian species in traditional food markets - infection prevention and control. *In**Science*, *371*(6525). 10.1126/science.abe5901. (American Association for the Advancement of Science).

[CR60] WHO. (2024). *Food safety*. https://www.who.int/news-room/fact-sheets/detail/food-safety. Accessed 24 Nov 2024.

[CR61] Wongprawmas, R., & Canavari, M. (2017). Consumers’ willingness-to-pay for food safety labels in an emerging market: The case of fresh produce in Thailand. *Food Policy,**69*, 25–34. 10.1016/j.foodpol.2017.03.004

[CR62] Xiu, C., & Klein, K. K. (2010). Melamine in milk products in China: Examining the factors that led to deliberate use of the contaminant. *Food Policy,**35*(5), 463–470. 10.1016/j.foodpol.2010.05.001

[CR63] Yuan, Y., Si, Z., Zhong, T., Huang, X., & Crush, J. (2021). Revisiting China’s supermarket revolution: Complementarity and co-evolution between traditional and modern food outlets. *World Development,**147*, 105631. 10.1016/J.WORLDDEV.2021.105631

[CR64] Zanatta, J. A. A. C., Fidelis, R., & Sakanaka, L. S. (2023). Method for selecting certification standards for food safety. *Food Security,**15*(4), 1071–1085. 10.1007/S12571-023-01370-8/METRICS

[CR65] Zhong, S., Crang, M., & Zeng, G. (2020). Constructing freshness: The vitality of wet markets in urban China. *Agriculture and Human Values,**37*(1), 175–185. 10.1007/S10460-019-09987-2/METRICS

